# Unveiling Microbial Dynamics in the Spontaneous Fermentation of Oat and Rice Okara Sourdoughs

**DOI:** 10.3390/foods15142442

**Published:** 2026-07-09

**Authors:** Federica Meanti, Paolo Bellassi, Alessandra Fontana, Margherita Dall’Asta, Annalisa Rebecchi

**Affiliations:** 1 Department for Sustainable Food Process (DiSTAS), Università Cattolica del Sacro Cuore, Via Stefano Leonida Bissolati 74, 26100 Cremona, Italy; federica.meanti1@unicatt.it (F.M.); paolo.bellassi@unicatt.it (P.B.); annalisa.rebecchi@unicatt.it (A.R.); 2 Department of Animal Science, Food and Nutrition (DiANA), Università Cattolica del Sacro Cuore, Via Emilia Parmense 84, 29122 Piacenza, Italy; margherita.dallasta@unicatt.it

**Keywords:** oat okara, rice okara, microbial succession, metagenomics, sourdough fermentation

## Abstract

Sourdough fermentation is increasingly explored as a sustainable strategy for the valorisation of cereal-based by-products, although okara from oat- and rice-based beverage production remains largely underexplored. This study investigates the microbial evolution and nutritional characteristics of oat and rice okara sourdoughs obtained by spontaneous fermentation using the back-slopping technique. High-throughput sequencing revealed dynamic but matrix-dependent microbial composition. At the beginning of fermentation, oat okara was dominated by the *Bacillus* genus, while the *Streptococcus* genus was the most abundant in rice okara. After 30 days of back-slopping, the bacterial communities of both matrices were dominated by *Lactobacillus*, accounting for 80.1% and 73.3% of the relative abundance in oat and rice okara sourdoughs, respectively. Secondary bacterial taxa differed between matrices, with *Weissella* prevailing in oat okara (7.0%) and *Acetobacter* in rice okara (11.2%). Yeast communities showed a substrate-dependent temporal succession, being initially dominated by Pichia in both oat and rice okara sourdoughs (96.6% and 97.1%, respectively), whereas Saccharomyces became predominant at later fermentation stages, reaching 54.8% in oat okara and 83.5% in rice okara. From a nutritional perspective, okara sourdoughs exhibited promising characteristics, being rich in proteins and free amino acids, particularly glutamic acid, aspartic acid and leucine. The fatty acid profile was marked by oleic, linoleic and stearic acids, while nutritionally important minerals associated with musculoskeletal and immune function, such as calcium, zinc and selenium, were present in relevant quantities in the sourdoughs. These findings provide new insights into oat and rice okara sourdoughs and support the use of fermented okara as a sustainable ingredient with potential functional relevance.

## 1. Introduction

In recent years, cereal-based products have been reformulated due to the growing trend towards healthy, low-processed and sustainable food consumption. This inclination has led both consumers and industry to become increasingly interested in the consumption and production of baked foods using sourdough. The fermentation of cereals to produce sourdough has been the technology used since antiquity [1,2]. Sourdough is a mixture of flour and water fermented by naturally occurring lactic acid bacteria (LAB) and yeasts, representing a dynamic microbial ecosystem in which community composition evolves over time [3,4], particularly during back-slopping. Many factors can influence this synergy: the type and origin of flour, the environment, process parameters, such as temperature and refreshment time, and interactions between microorganisms. These microbial interactions play a key role in improving the nutritional, sensory and technological quality of bakery products [5,6,7,8,9,10].

In recent years, increasing attention has been paid to by-products from the production of plant-based beverages [11]. Much of the literature, however, has evaluated the reuse of soy okara alone [12], while studies on residues from oat- and rice-based drinks are very limited, particularly in the context of sourdough fermentation. Soy okara can be exploited either in its original form or it can undergo further processing to obtain flour. In both forms, it is incorporated into foods to enhance nutritional and functional properties, e.g., beverages and baked goods [13,14]. Several studies have reported that the nutritional quality of soy okara, as well as its health benefits, are improved by the fermentation technique [15,16,17]. The release of hydrolytic enzymes during fermentation promotes the breakdown of complex macromolecules, such as lipids, proteins and fibres, into smaller and more bio-accessible compounds. Okara, therefore, represents a great potential as a functional ingredient with positive health effects and prebiotic properties [18,19]. The by-products of the production of vegetable drinks from rice and oats have a high-protein content and essential amino acids. Rice okara has a protein value of 57.28 ± 1.32 g/100 g and oat okara has a protein value of 35.38 ± 0.76 g/100 g compared to soy okara which has a protein value of 27.42 ± 10.11 g/100 g [20]. Some authors have suggested some uses of oat okara. Bartkiene et al. [21] claimed that 15% of fermented oat okara improves the quality and nutritional value of wheat bread. Esposito et al. [20], comparing the composition of rice okara with that of soy, assume that it could be used to produce meat analogues, thanks to its technological characteristics and the fact that its proteins are hypoallergenic and can be consumed even by those who suffer from gluten allergy.

The aim of this study was therefore to investigate the microbial composition and dynamics of oat- and rice-based okara sourdoughs during fermentation carried out using the back-slopping technique. Furthermore, the nutritional characterisation was specifically directed towards free amino acids and selected minerals (i.e., calcium, zinc and selenium), given their importance for musculoskeletal function and immune system support. This integrated approach aims to contribute to the development of healthier and more sustainable food products, in line with the principles of the circular economy.

## 2. Materials and Methods

### 2.1. Sourdough Preparation

Packtin srl (Reggio Emilia, Italy) provided oat and rice okara flours used in this study. The flour is produced by removing moisture from the okara of the cereal and then freezing, drying, and grinding it. According to the nutritional information declared by the manufacturer, the oat okara flour is characterised by an energy value of 332 kcal, with 3.93 g/100 g of fat (of which 0.71 g/100 g saturated), 19.50 g/100 g of carbohydrates (of which 13.20 g/100 g sugars), 22.70 g/100 g of dietary fibre and 43.30 g/100 g of protein. The rice okara flour shows an energy value of 358 kcal, with 1.40 g/100 g of fat (including 0.30 g/100 g saturated fat), 16.90 g/100 g of carbohydrates (of which 13.40 g/100 g sugars), 6.30 g/100 g of dietary fibre and 66.30 g/100 g of protein.

The flour samples were used to produce the oat okara sourdough (OOS) and the rice okara sourdough (ROS) with only warm tap water (35 ± 5 °C) added and the blend was prepared in a 2:1 water-to-flour proportion. Doughs were prepared in triplicate. All ingredients were mixed with a TK20 kneading machine (Tekno Stamap, Vicenza, Italy) for 12 min and kept at room temperature (25 ± 2 °C) for 24 h, allowing for spontaneous fermentation. Doughs were refreshed daily over a period of 30 days. At each refreshment, 60 g of fermented dough were mixed with 40 g of water and 20 g of flour, to maintain consistent fermentation conditions. Samples were collected at 0 day (t0), 1 day (t1), and 30 days (t2) after refreshment.

The pH values of OOS and ROS at the different sampling times (t0, t1, t2) were measured in triplicate with a HANNA pH metre HI-2202 Edge^®^blu (Hanna Instruments, Padova, Italy).

### 2.2. DNA Extraction, Amplicon Sequencing, and Bioinformatic Analysis

DNA extraction was performed from 200 mg of each sample using the Fast DNA^TM^ SPIN Kit for Soil (MP Biomedicals, LLC, Solon, OH, USA) following the manufacturer’s protocol. DNA quantity and purity were assessed using Qubit (Invitrogen, Carlsbad, CA, USA) fluorometry and agarose gel electrophoresis, and then sent to the sequencing facility for 16S rRNA gene (V3–V4 region) [22] and ITS rRNA gene (ITS1 region) [23] amplicon sequencing using the Illumina Miseq technology (2 × 250 bp) (Illumina Inc., San Diego, CA, USA).

Raw sequence data were processed using QIIME2 v2024.5 [24]. Demultiplexing and quality filtering were performed using the q2-demux plugin, followed by denoising with Deblur [25]. The V3–V4 (16S rRNA gene) and ITS data sets were analysed separately. Taxonomic assignment of bacterial ASVs was performed using the GreenGenes2 database [26], while fungal (yeast) ASVs were classified using the UNITE database. ASV count tables were imported into Rv.4.4.2 (R Core Team, Vienna, Austria, 2023) and analysed using the phyloseq package (v.1.50.0). Samples were rarefied to an even sequencing depth corresponding to the minimum library size observed across samples (i.e., the sample with the lowest number of reads), using the rarefy_even_depth function to account for differences in library size. Alpha diversity was estimated using the Shannon index (vegan package v.2.7.1), and differences among groups were explored using ANOVA followed by pairwise Welch’s *t*-tests with false discovery rate (FDR) correction (FDR < 0.05). Beta diversity was calculated using Bray–Curtis dissimilarity, and ordination was performed via the Principal Coordinates Analysis (PCoA) using the ape package (v.5.8.1). Differences in community composition were tested using PERMANOVA (adonis2, vegan package v.2.7.1) with permutations. Pairwise PERMANOVA comparisons were conducted using the pairwiseAdonis package (v.0.4.1) with FDR correction (FDR < 0.05).

In addition, a single-factor analysis was performed to assess changes in the main microbial genera shared across the matrix over fermentation time. Statistical significance was explored using Fisher’s exact test, followed by FDR correction for multiple comparisons (FDR < 0.05). To focus on biologically relevant taxa, only genera with a relative abundance of at least 2% in at least one of the two compared time points were included in the analysis. The magnitude of these changes was quantified using fold change (FC) analysis.

### 2.3. Chemical and Nutritional Analyses

Amino acid composition was determined by a certified external laboratory (Eurofins Vitamin Testing Denmark A/S, Vejen, Denmark). Total amino acids were analysed after acid hydrolysis according to ISO 13903:2005 [27] using ion chromatography with UV detection (IC-UV; method DI004). Cystine and methionine were determined after oxidative hydrolysis following ISO 13903:2005 (method DJ011, IC-UV), while tryptophan was analysed according to EU Regulation 152/2009 using liquid chromatography with fluorescence detection (LC-FLD). Results were expressed as g/100 g of dry matter.

Mineral content was determined by an accredited external laboratory (Eurofins Chemical Control Srl, Cuneo, Italy). Calcium, zinc and selenium were analysed by inductively coupled plasma mass spectrometry (ICP-MS) according to method MI 2385 rev. 07/2022. Results were expressed as mg/kg of dry matter.

Nutritional composition including ash, protein, total dietary fibre, moisture, total fat, fatty acid profile, sugars, energy content and total carbohydrates was determined by Eurofins Chemical Control Srl (Italy) using standard official methods. Ash and protein were determined according to DM 27/05/1985 (GU n°145 21/06/1985), combined with AOAC 992.23 (1998) and ISTISAN 1996/34 [28]. Total dietary fibre (high molecular weight) was determined enzymatically and gravimetrically following AOAC 991.43 (2000). Moisture and total fat were measured by gravimetric methods (DM 27/05/1985; MI 2405 rev. 03/2023). Saturated, monounsaturated, polyunsaturated and trans fatty acids were quantified by calculation and fatty acid profiles (% of methyl esters) were determined by gas chromatography with flame ionisation detection (GC-FID; MI 2342 rev. 05/2023). Sugars were analysed using high-performance anion-exchange chromatography with pulsed amperometric detection (HPAEC-PAD; MI 1533 rev. 08/2022). Energy content and total carbohydrates were calculated according to standard European and AOAC methods. All results were expressed per 100 g of dry matter. Since the analyses were carried out by an external laboratory, it was not possible to perform statistical analysis on the chemical and nutritional data, as the sample’s replicates were not available. The data are expressed as means and standard deviations.

## 3. Results and Discussion

### 3.1. Sourdough’s pH Variations

The pH values recorded during fermentation showed a progressive acidification of both oat and rice okara sourdoughs (Table 1). In oat okara sourdough (OOS), pH decreased from 5.18 ± 0.02 at t0 to 4.50 ± 0.01 at t1, followed by a slight decrease at t2 (4.45 ± 0.10). A similar trend was observed in rice okara sourdough (ROS), where pH decreased from 6.44 ± 0.01 at t0 to 5.20 ± 0.04 at t1 and further declined to 4.90 ± 0.01 at t2. Overall, fermentation resulted in lower pH values in both matrices, consistent with organic acid production during sourdough fermentation. The observed pH decrease is consistent with the acidification of sourdough systems during fermentation [29].

### 3.2. Microbial Diversity

**Figure 1:**
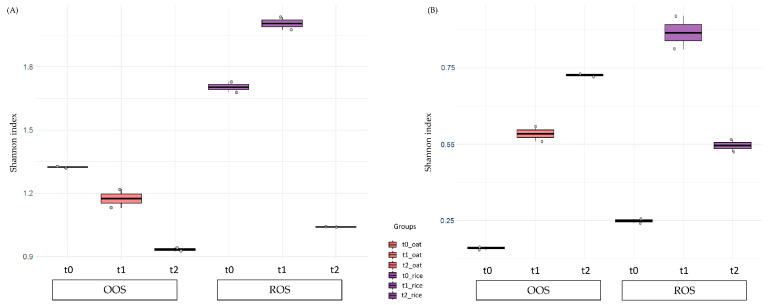
Dynamics of microbial α-diversity (Shannon index) for (**A**) bacterial and (**B**) yeast communities during the fermentation of oat and rice okara sourdoughs at 0 days (t0), 1 day (t1) and 30 days (t2) of fermentation.

Explorative analysis of Shannon α-diversity of microbial community (bacteria and yeasts) across the different fermentation stages of okara and rice sourdough (Figure 1) revealed a general decline in microbial diversity from the onset of fermentation to the end of the back-slopping process, indicating a progressive simplification of the community structure. Nevertheless, distinct trends were evident between the rice-based (ROS) and oat-based (OOS) sourdoughs. The observed differences in microbial composition at t0 between oat and rice okara likely reflect the distinct initial microbiota naturally associated with the different plant matrices, which may contribute to the matrix-dependent microbial succession observed during fermentation.

In OOS samples (Figure 1A), Shannon diversity of bacteria decreased gradually throughout fermentation, displaying a nearly linear trend. A slight increase at t1 compared to t0 and t2 suggested the transient emergence of additional taxa after 24 h of fermentation. By t2 compared to t0, diversity decreased, suggesting the establishment of a more stable and less complex microbial community dominated by a limited number of adapted species. In agreement with previous studies [30,31,32], our data indicate that sourdough ecosystems experience an early stage of high microbial diversity, which gradually declines as fermentation and successive back-slopping promote the dominance of a few well-adapted, acidophilic taxa.

In contrast, ROS samples exhibited overall higher Shannon diversity than OOS in the different fermentation times. Interestingly, diversity increased from t0 to t1, reflecting a temporary enrichment in species diversity during the initial fermentation phase. The diversity values remained comparable between t0 and t1 but dropped substantially at t2, consistent with the selective stabilisation of the microbial community characteristic of mature sourdough ecosystems. PCoA based on β-diversity metrics accounted for approximately 72.75% of the total variance (PCoA1 = 43.99%; PCoA2 = 28.58%) and revealed statistically significant differences in microbial community composition (*p*-value = 0.001) (Figure S1). Distinct clustering patterns were observed, with ROS t0 samples forming a separate group from all other time points. ROS t1 samples clustered closely with OOS t0 and t1 samples, indicating convergence of microbial community structures during early stages. At t2, ROS and OOS samples exhibited highly similar microbial profiles that were clearly segregated from earlier time points and matrices, suggesting a temporal stabilisation and maturation of the microbial communities. Considering the compositional differences in the initial matrices, the dominance of specific bacterial genera at t2 likely reflects selective pressures imposed by technological conditions that favoured the proliferation of specific taxa. Our results are consistent with those reported by McKenney et al. [30], who observed a decrease in Shannon α diversity during sourdough fermentation, reflecting the progressive simplification of microbial communities associated with the establishment of dominant taxa. However, transient increases in diversity at intermediate stages suggest that community dynamics are strongly influenced by the initial microbiota and matrix composition [31]. Such temporary rises may reflect the bloom of conditionally rare taxa that expand under specific fermentation conditions before being outcompeted by acid-tolerant and better-adapted species [32]. These findings highlight that changes in α-diversity during fermentation are context-dependent and shaped by both substrate characteristics and technological parameters.

Analysis of yeast α-diversity (Figure 1B) revealed trends markedly different from those observed for bacteria, with distinct dynamics emerging between oat-based (OOS) and rice-based (ROS) sourdoughs. In OOS samples, yeast diversity followed a pattern opposite to that of bacterial communities. At t0, OOS displayed very low yeast diversity and limited species variability, indicating a sparse and uneven initial yeast community. Diversity progressively increased throughout fermentation, reaching the highest values at t2, where the yeast community appeared both richer and more evenly distributed across taxa. ROS samples exhibited a trajectory more closely aligned with bacterial trends, although with yeast-specific characteristics. As in OOS, ROS showed low yeast abundance and limited variability at t0. At t1, ROS displayed a pronounced increase in both abundance and species variability; by t2, both abundance and diversity declined again, and in fact, evenness improved. PCoA based on β-diversity distances showed that the first two axes explained 99.73% of the total variance (PCoA1 = 56.88%; PCoA2 = 42.85%) and detected significant differences among yeast community structures (*p*-value = 0.001).

### 3.3. Back-Slopping Effect on Microbial Communities of Sourdough

#### 3.3.1. Bacterial Community

**Figure 2:**
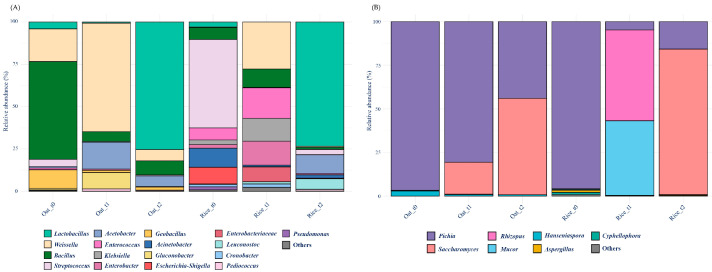
Relative abundance of bacterial, fungal and yeast genera during the fermentation of oat- and rice-based sourdoughs. Bar plots showing the relative abundance of the main bacterial (**A**) and fungal/yeast (**B**) genera detected in oat okara and rice okara sourdoughs at three fermentation stages (t0, t1, t2). Only genera with a relative abundance ≥ 1% are shown in the plots, while those below this threshold are collectively grouped as “Others”.

The taxonomic identification analysis at the three fermentation times was performed to identify changes in the microbial community during the production of a mature sourdough and to determine the main genera driving the fermentation in the two different types of flour. Figure 2 shows the bacterial genera found in the two sourdoughs. Specifically, OOS at the first fermentation step showed predominance of the genus *Bacillus* (57.82%), followed by *Weissella* (19.88%) and *Geobacillus* (10.79%) and, to a lesser extent, *Streptococcus* (4.27%) and *Lactobacillus* (3.96%). At t1, however, *Weissella* (64.25%) took over from *Bacillus* (5.65%), which decreased dramatically together with other genera, while two new genera appeared: *Acetobacter* (15.87%) and *Gluconobacter* (9.72%), which are reduced again at t2, especially the latter. At the end of fermentation, *Lactobacillus* (80.14%) was the dominant genus, followed by *Bacillus* (8.89%) and *Weissella* (6.97%).

ROS at the beginning of the fermentation (t0) showed *Streptococcus* (52.66%) as the dominant genus, followed by *Acinetobacter* (11.28%) and *Escherichia*_*Shigella* (10.09%). Interestingly, there was a very low presence of *Lactobacillus* and *Weissella*, while this latter at t1 was notable as the dominant genus (32.89%), followed by *Enterococcus* (21.37%), *Klebsiella* (15.96%), *Bacillus* (13.18%) and *Enterobacter* (9.50%). Similarly to what was observed for OOS, at the end of fermentation, the highly predominant genus was *Lactobacillus* (73.33%), followed by *Acetobacter* (11.21%) and also *Leuconosctoc* (6.54%) absent or present in low quantity in previous times.

Our findings highlight that in mature OOS and ROS, the genus that controlled the fermentation was *Lactobacillus*. In fact, this genus makes up the majority of the microorganisms isolated from sourdough and cereal fermentation [33]. Lactobacilli can have three different types of glucose metabolism (obligate homofermentants, obligate heterofermentants and facultative heterofermentants), thus influencing the development of the sourdough itself as well as the remaining microflora. The species of lactobacilli themselves that can develop in sourdough depend on several factors, such as the starting flour, the technology used and the production area [34]. In particular, obligate heterofermentative lactobacilli tend to be dominant in sourdough, as they are able to metabolise maltose through the 6-PG/PK pathway and also fructose, which acts as an electron acceptor. They are also able to grow at the pH and temperature that develop in sourdough, also due to their high adaptive capacity to environmental stresses by implementing different phenotypic responses. Finally, they are also capable of producing antimicrobial compounds [29]. Of particular interest is their proteolytic metabolism. Sourdough lactobacilli often do not exhibit the proteinase activity associated with the cell wall [35,36]. It has been observed that the acidification carried out by lactic acid bacteria favours the activation of aspartate-proteinases of the cereals themselves, resulting in the release of oligopeptides that are transported and degraded within the bacterial cell [37,38]. The intracellular peptidases of lactic bacteria then allow the release of peptides and free amino acids in the dough, even higher than that of a chemically acidified dough [39,40]. There is then a primary proteolysis, through the endogenous proteases of cereals that allow the release of oligopeptides, and a secondary proteolysis, carried out by lactic bacteria that allows the release of free amino acids and small peptides, which are also precursors of aromatic agents [37]. In direct comparison, the two sourdoughs showed a partly overlapping bacterial trajectory that was nonetheless strongly influenced by the starting matrix. In both cases, *Lactobacillus* was scarcely represented at the onset of fermentation, decreased further at t1 and, ultimately, became predominant in the mature sourdoughs (80.14% in OOS and 73.33% in ROS). *Weissella* showed in OOS and ROS different abundance at t0 and t2, while it became dominant at t1 in both matrices. The trends of the remaining genera differed more markedly between the two matrices. *Acetobacter* was abundant in OOS at t1, whereas in ROS it became prominent only at t2. The occurrence and temporal enrichment of *Acetobacter* in both matrices is consistent with previous reports describing the involvement of acetic acid bacteria in sourdough ecosystems, where they can persist during refreshment phases and interact metabolically with yeasts and lactic acid bacteria [41,42,43]. By contrast, *Gluconobacter* was relevant only in OOS. The detection of this genus, predominantly in oat okara sourdough, is in agreement with its well known oxidative metabolism of sugars and organic acids, which supports its presence in oxygen exposed and carbohydrate rich fermented matrices [44,45]. In particular, acetic acid bacteria, such as *Gluconobacter* are known to grow and remain metabolically active at low pH values, including around pH 4.5, as observed in oat okara at the early fermentation stage (t1) [46,47].

The *Leuconostoc* genus was virtually absent in OOS but detected in ROS especially at the end of fermentation. This distribution might suggest that *Leuconostoc* prefers initial fermentation environments characterised by a higher pH and easily fermentable carbohydrates, conditions that are more likely to be met by rice okara than by oat okara. In contrast, oat okara is comparatively richer in dietary fibre and structurally complex polysaccharides, which may limit the establishment of this genus [48,49]. *Bacillus* also exhibited distinct dynamics, being abundant in OOS at t0 and t2, while in ROS it was mainly observed at t2. This behaviour is consistent with the frequent association of the *Bacillus* species with cereal-based substrates and food fermentations, as well as their metabolic versatility across different food matrices [50]. *Streptococcus*, in both sourdoughs, is the most abundant at t0, then decreases rapidly, in agreement with Bartkiene et al. [51] reporting *Streptococcus* as a species isolated during early spontaneous sourdough fermentation phases. In both matrices several potentially undesirable genera were detected at early fermentation stages, including *Geobacillus* in OOS and *Acinetobacter*, *Escherichia*/*Shigella*, *Enterobacter* and *Klebsiella* in ROS. These taxa were markedly reduced at t2, concomitantly with the establishment of a *Lactobacillus*-dominated community and the pH reduction. Overall, these findings indicate that genus-level community composition during fermentation was strongly shaped by the initial matrix.

#### 3.3.2. Fungal and Yeast Community

Overall, a few genera were detected at different timepoints in OOS and ROS. In the case of OOS, at the start of the fermentation, *Pichia* (96.63%) was the dominant genus, followed by *Hanseniaspora* (2.99%). After 24 h, it decreased at 80.08% and 0.96% respectively. The *Saccharomyces* was detected at 18.79% at the end of fermentation it took over from the other two genera (54.79%), although *Pichia* (44.54%) remained abundant. At the beginning of the fermentation process, also in ROS *Pichia* (97.14%), was the predominant yeast, followed in very small quantities by other genera, while at time t1, it decreased drastically (4.88%), giving way to *Rhizopus* (51.94%) and *Mucor* (42.72%). As observed in OOS, at t2, *Saccharomyces* (83.75%) appeared as the dominant genus followed by *Pichia* (15.47%).

Comparing ROS and OOS, it could be seen that *Pichia* and *Saccharomyces* had roughly the same trend: *Pichia* was the genus that activated fermentation, and *Saccharomyces* was the one that carried it forward into the following back-slopping phases. The two mixtures differed in the other eukaryotic communities identified in the samples; in particular, in the OOS, the genus *Hanseniaspora* was found as another fermentation actor, while in ROS, the genus *Rhizopus* was more abundant as a fermentation agent.

Our results are consistent with Deak and Beuchat et al. [52] and De Vuyst and Vancanneyt et al. [53] who described these three genera among the most isolated in the sourdough. In particular, the species *Saccharomyces cerevisiae* is less frequently found in flours at the beginning of fermentation, but is more easily identifiable at later stages. The production of dough seems to be precisely the condition that favours its growth [52]. The high presence of the genus *Pichia* is very interesting, as they have been recognised as important contributors to the flavour of certain fermented foods. Some species have been shown to be responsible for the development of fruity, green-herb and cheese flavours [54], others have been strongly correlated with total volatile esters [55]. Furthermore, it has been observed that some species are able to enhance the activity of specific *Lactobacillus* species during co-fermentation, resulting in increased production of alcohol acetates and fatty acid ethyl esters [56]. Indeed, fermentation of plant products in the presence of *Pichia* spp. is positively correlated with aromatic compounds, such as 4-ethylphenol, phenyl alcohol and methyl salicylate [57].

### 3.4. Oat and Rice Okara Sourdough Fermentation Characterising Genera (Single-Factor Analysis)

Sourdough fermentation is sustained by complex microbial consortia in which lactic acid bacteria and yeasts coexist, and genera such as *Lactobacillus*, *Weissella*, *Acetobacter*, *Saccharomyces* and *Pichia* are widely recognised as key microbial players shaping the acidification dynamics, metabolic activity and overall stability of sourdough ecosystems [58,59].

**Figure 3:**
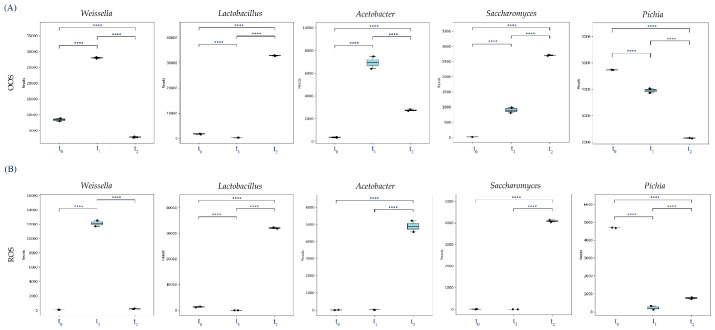
Single factor metagenomic boxplots of key fermentation genera. Read abundance of *Lactobacillus*, *Weissella*, *Acetobacter*, *Saccharomyces* and *Pichia* at t0, t1 and t2 in oat (**A**) and rice (**B**) okara sourdoughs. All comparisons tested were highly significant (**** *p* < 0.0001).

At the early stage of fermentation (t1 vs. t0), both OOS and ROS showed significant yet substrate-dependent microbial shifts (Figure 3).

The *Acetobacter* genus increased markedly in OOS (log_2_FC = 4.25), while in ROS, it was consistently lower, confirming that the oat substrate provided more favourable conditions for acetic acid bacteria proliferation [60]. *Weissella* genus, conversely, showed a stronger response in ROS (log_2_FC = 6.83) compared with OOS (log_2_FC = 1.73), highlighting that this genus dominates the early stages of rice okara fermentation. *Weissella* has been repeatedly isolated from rice-based fermented matrices, including fermented rice grains [61] and traditional rice wine koji [62], indicating that rice provides a highly suitable substrate for the establishment and early dominance of this genus. At the same early stage, *Lactobacillus* showed negative fold changes in both matrices (OOS log_2_FC = −2.51; ROS log_2_FC = −7.17), indicating a pronounced temporary reduction during the initial microbial succession phase.

At the t1 vs. t0 comparison, the two substrates showed distinct yeast dynamics. In OOS, genera such as *Saccharomyces* increased sharply (log_2_FC = 5.79), while *Pichia* decreased slightly, indicating the former as the main driver of the fermentation. In contrast, ROS showed a significant decrease for both yeast genera. At the later fermentation stage (t2 vs. t1), both sourdoughs were characterised by the dominance of lactic acid bacteria and the consolidation of acetic acid bacteria and fermentative yeasts. The *Acetobacter* genus has recorded a stronger enrichment in ROS (log_2_FC = 8.17) than in OOS, confirming that this genus could also be present in prolonged fermentations and be part of mature sourdoughs [63]. *Lactobacillus* was significant in both sourdoughs, presenting high values between OOS (log_2_FC = 6.73) and ROS (log_2_FC = 11.80), indicating the establishment of a stable lactic acid bacterial core community typical of mature sourdough ecosystems. This aligns with literature reports showing that, despite temporal variability in the bacterial community, *Lactobacillus* remains widely present and dominant. Over 60 different species have been identified, highlighting the genus diversity within sourdough ecosystems. Although the specific species composition may vary, *Lactobacillus* consistently constitutes the predominant group, confirming a functionally stable core community [7]. At the later fermentation stage (t2 vs. t1), *Weissella* showed a strongly significant decrease in both matrices, reflecting a progressive decline of this genus during the later stages of fermentation. *Weissella* is commonly associated with spontaneous fermentations and is frequently described as a transient member of sourdough microbial communities, being less competitive during advanced fermentation stages compared to more acid-tolerant lactobacilli [64]. In addition, substrate composition plays a key role in shaping its persistence, as reported by Wolter et al. [65], who observed a strong substrate dependence of *Weissella* growth. Oat-based matrices, being relatively poor in readily fermentable sugars, may limit the competitiveness of *Weissella* during prolonged fermentations, thereby contributing to its progressive decline.

Within the yeast community, *Saccharomyces* displayed a strong enrichment in ROS, reaching exceptionally high values (log_2_FC = 11.41), while a lower enrichment, but still significant, was observed in OOS (log_2_FC = 1.56). This pattern reflects the progressive dominance of this genus at advanced fermentation stages, which is typically associated with the accumulation of ethanol and other stress-related metabolites that select for more tolerant yeast species [66].

### 3.5. Nutritional Characteristics of Oat and Rice Okara Across Fermentation

During sourdough fermentation, the metabolic activity of lactic acid bacteria and yeasts lead to some biochemical transformations in the dough matrix. The following sections present the main biochemical and nutritional changes that were observed between the flour and the corresponding sourdough, supported by quantitative data. These results should be interpreted as indicative trends rather than statistically validated differences.

#### 3.5.1. Nutritional Composition Table

Overall, the nutritional composition of oat and rice okara sourdoughs remained comparable to that of the corresponding flours, with the exception of carbohydrate (Table 2).

In oat okara, energy values were within a similar range for flour and sourdough (395.38 vs. 380.95 kcal/100 g). Total fat was comparable (14.41 ± 1.10 vs. 14.21 ± 1.94 g/100 g), as were saturated fats (2.44 ± 0.51 vs. 2.51 ± 0.91 g/100 g). In rice okara, energy values were similar between flour and sourdough (399.27 vs. 390.19 kcal/100 g). Total fat remained close (5.34 ± 0.65 vs. 5.80 ± 1.19 g/100 g), as did saturated fats (1.44 ± 0.38 vs. 1.64 ± 0.71 g/100 g).

Carbohydrate content in rice okara flour was about twice that of the oat okara flour (16.92 and 7.96 g/100 g respectively) and was strongly reduced in both sourdoughs, (<0.5 and 5.76 g/100 g respectively), while sugars were <0.5 g/100 g in both okara sourdoughs, in line with their use by microorganisms in performing fermentation processes. Protein content, measured as total nitrogen (N × 6.25) present in the sample, of oat and rice okara sourdoughs showed a slight upward trend reaching 49.22 g/100 g in oat and 74.02 g/100 g, respectively; as well as dietary fibre showed a slight increase during fermentation, from 25.96 to 28.19 g/100 g in OOS and from 5.89 to 8.66 g/100 g in ROS. These observations are consistent with established features of cereal fermentations, in which fermentable carbohydrates are depleted, while protein and dietary fibre can appear relatively enriched on a Dry Matter Basis (DMB) read-out and/or be affected by microbial biomass and nitrogen turnover [67,68,69].

#### 3.5.2. Amino Acid Profile

**Figure 4:**
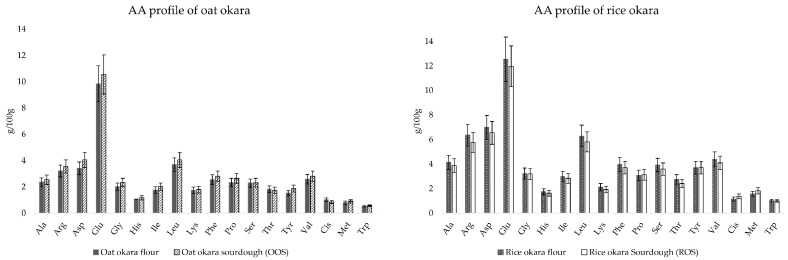
Free amino acid content in flour and sourdough samples (g/100 g), values are expressed on a dry matter basis (Dry Matter Basis, DMB). Values are expressed as mean ± standard deviation.

Figure 4 and Table 3 summarise the free amino acid content of oat and rice okara flours and sourdoughs.

Despite slight variations in mean values, the fermentation process did not negatively affect the high amino acid content of okara flours, suggesting that sourdough fermentation preserves their nutritional potential. In oat okara, most amino acids appeared slightly higher in the sourdough than in the flour, whereas the opposite trend was observed in rice okara. In particular, across both matrices, glutamic acid was the most abundant free amino acid, followed by aspartic acid and leucine (Figure 4).

Glutamic acid is a well-known compound that plays a key role in the development of umami taste and in shaping the aromatic profile of naturally fermented baked products [70]. This finding was supported by studies describing the formation, during sourdough fermentation, of peptides and glutamate derivatives with relevant sensory activity, which contributed to flavour complexity [71]. In this context, glutamate has often been associated with the sensory attributes of mature sourdoughs, including flavour rounding and enhancement of overall flavour [72,73].

Aspartic acid is a metabolically relevant amino acid involved in major biochemical pathways, including amino acid interconversion and energy metabolism [74]. From a food perspective, aspartic acid has been recognised as an important contributor to flavour balance and taste perception in fermented foods. In particular, aspartic acid is associated with buttery flavours, supporting overall sensory complexity beyond volatile aroma compounds [75].

Moreover, leucine is a nutritionally essential branched chain amino acid (BCAA) and one of the most abundant amino acids in high quality protein sources. Leucine plays a central regulatory function in protein and energy metabolism. In particular, leucine is widely recognised as a pivotal regulator of skeletal muscle protein synthesis and muscle mass maintenance [76]. The presence of leucine, therefore, represents an important nutritional attribute in protein rich fermented ingredients, supporting their potential application in the development of foods with enhanced nutritional value.

#### 3.5.3. Fatty Acid Composition

**Figure 5:**
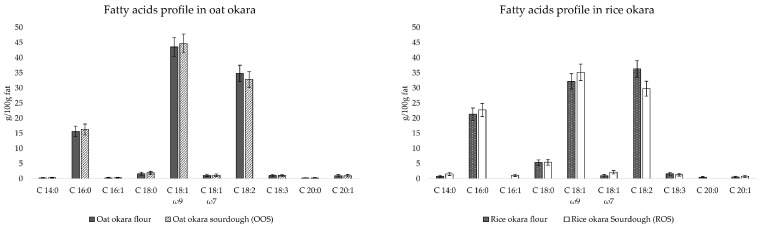
Fatty acid content in flour and sourdough samples (g/100 g fat). Values are expressed as mean ± standard deviation.

The fatty acid profiles of oat and rice okara showed comparable patterns between flour and sourdough samples (Figure 5).

In both matrices, fermentation did not result in marked changes in the relative distribution of fatty acids, with values remaining within a similar range. In oat okara flour and sourdough, oleic acid (C18:1 ω9) was the predominant fatty acid, followed by linoleic acid (C18:2) and palmitic acid (C16:0), accounting for the largest proportion of the lipid fraction. Similar results were reported by Gu et al. [77] for oat flour. In rice okara, linoleic acid was slightly more abundant than oleic acid in the flour, whereas in the sourdough, the ratio was reversed. A comparable fatty acid profile in rice flour was reported by Samaranayake et al. [78]. Minor fatty acids, including saturated and long-chain species, were present at low levels and did not show substantial variation following fermentation. Overall, these results indicate that the fermentation in the sourdough process has no effect on the fatty acid composition under the conditions applied.

From a nutritional perspective, the predominance of unsaturated fatty acids, such as oleic and linoleic acids, is of particular interest, as these lipids have been widely associated with favourable metabolic effects and with the modulation of inflammatory and immune-related processes. Oleic acid, a major monounsaturated fatty acid, has been consistently linked to anti-inflammatory and cardiometabolic benefits, which are considered key factors in promoting healthy ageing [79,80]. Linoleic acid, an essential polyunsaturated fatty acid, plays a crucial role in membrane structure and function and is involved in immune and inflammatory responses [81]. Altogether, the prevalence of unsaturated fatty acids supports the nutritional relevance of okara derived lipid fractions in the context of dietary strategies aimed at maintaining metabolic and immune health during ageing.

#### 3.5.4. Mineral Content

**Figure 6:**
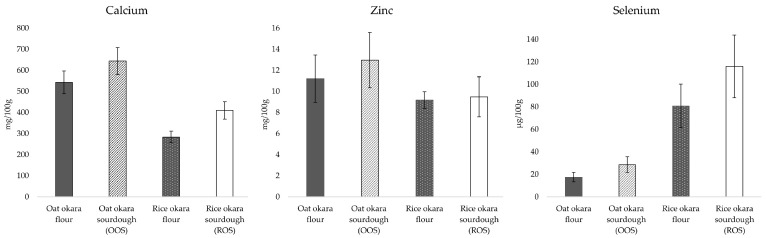
Mineral content in flour and sourdough samples (mg/100 g for calcium and zinc; µg/100 g for selenium). Values are expressed on a dry matter basis (Dry Matter Basis, DMB). Values are expressed as mean ± standard deviation.

Calcium, zinc and selenium were selected as target minerals because they are considered essential for the maintenance of bone tissue and for supporting the immune system. Fermentation was associated with an overall rise in the measured levels of these minerals, as shown in Figure 6.

In particular, OOS exhibited higher levels of calcium and zinc, whereas ROS was especially rich in selenium.

Calcium plays a fundamental role in the maintenance of bone structure and skeletal integrity, which is particularly critical in older adults due to the increased risk of bone loss and fragility [82]. Zinc is an essential trace element involved in numerous enzymatic and regulatory processes and is recognised for its pivotal role in supporting immune function; inadequate zinc status has been associated with immune senescence and increased susceptibility to infections during ageing [83]. Selenium, through its incorporation into selenoproteins, contributes to antioxidant defences and immune regulation, helping to counteract oxidative stress and low-grade chronic inflammation that commonly accompany the ageing process [84]. Together, these minerals are widely regarded as important contributors to musculoskeletal health and immune competence in ageing populations.

Furthermore, fermentation may have created conditions potentially favouring mineral bioaccessibility, although specific bioavailability assays would be required to confirm this effect. This hypothesis is supported by the fact that cereal- and legume-derived matrices may contain antinutritional factors, such as phytic acid and tannins, which can chelate minerals and reduce their intestinal absorption. Microbial fermentation has been reported to contribute to the degradation of these compounds, potentially increasing the fraction of minerals available for nutritional uptake. Therefore, while the mineral profile observed in okara-based sourdoughs is nutritionally relevant, further studies are needed to determine whether fermentation effectively improves mineral bioaccessibility and bioavailability in these matrices [85,86].

## 4. Conclusions

This study investigated the microbial and nutritional characteristics of sourdoughs produced from oat and rice okara flours. Fermentation induced a marked time-dependent restructuring of bacterial and yeast communities, ultimately leading to the establishment of microbial profiles consistent with mature sourdough ecosystems. Although substrate-specific differences were observed during the early and intermediate fermentation stages, the microbial communities progressively converged towards comparable mature ecosystems, suggesting that the selective pressures imposed by sourdough fermentation may partly override the initial matrix-dependent differences.

From a nutritional perspective, fermentation preserved the protein, amino acid, and fatty acid contents of the okara flours, while contributing to a reduction in carbohydrate levels. The retention of nutritionally relevant amino acids and essential fatty acids, together with the presence of minerals such as calcium, zinc, and selenium, supports the potential of okara-based sourdoughs as sustainable ingredients for food formulation. However, as the nutritional analyses were not supported by biological replication or statistical comparison, the observed compositional changes should be interpreted as indicative trends rather than definitive fermentation-induced effects.

These findings support the feasibility of valorising oat and rice okara through sourdough fermentation and provide preliminary evidence for its potential application in the upcycling of cereal-based by-products. This approach may contribute to the development of fermented ingredients with distinctive technological and sensory properties, while maintaining nutritional quality and supporting broader strategies aimed at improving the sustainability of food systems.

## Figures and Tables

**Table 1:** pH values measured during oat and rice okara sourdough fermentation. pH values are reported for oat okara sourdough (OOS) and rice okara sourdough (ROS) at three fermentation stages (t0, t1, t2) and are expressed as mean ± standard deviation.

Sample	pH
OOS t0	5.18 ± 0.02
OOS t1	4.50 ± 0.01
OOS t2	4.45 ± 0.10
ROS t0	6.44 ± 0.01
ROS t1	5.20 ± 0.04
ROS t2	4.90 ± 0.01

**Table 2:** Nutritional values are expressed on a dry matter basis (Dry Matter Basis, DMB). Energy was calculated according to the criteria established by Regulation (EU) No. 1169/2011. Values are expressed as mean ± standard deviation.

Sample	Energy (kcal)	Fat (g/100 g)	Of Which Saturated (g/100 g)	Carbohydrate (g/100 g)	Of Which Sugars (g/100 g)	Protein (g/100 g)	Fibre (g/100 g)
Oat okara flour	395.38	14.41 ± 1.10	2.44 ± 0.51	7.96	6.80 ± 1.27	45.55 ± 4.12	25.96 ± 3.65
Oat okara sourdough (OOS)	380.95	14.21 ± 1.94	2.51 ± 0.91	<0.5	<0.5	49.22 ± 4.53	28.19 ± 6.02
Rice okara flour	399.27	5.34 ± 0.65	1.44 ± 0.38	16.92	12.17 ± 1.90	67.91 ± 6.14	5.89 ± 1.43
Rice okara Sourdough (ROS)	390.19	5.80 ± 1.19	1.64 ± 0.71	5.76	<0.5	74.02 ± 1.72	8.66 ± 2.82

**Table 3:** Free amino acid content of oat and rice sourdoughs (g/100 g), values are expressed on a dry matter basis (Dry Matter Basis, DMB). Values are expressed as mean ± standard deviation.

Amino Acid (g/100 g)	Oat Okara Flour	Oat Okara Sourdough (OOS)	Rice Okara Flour	Rice Okara Sourdough (ROS)
Ala	2.353 ± 0.327	2.537 ± 0.354	4.140 ± 0.578	3.865 ± 0.557
Arg	3.202 ± 0.447	3.554 ± 0.499	6.352 ± 0.885	5.760 ± 0.818
Asp	3.409 ± 0.479	4.038 ± 0.571	6.987 ± 0.975	6.540 ± 0.929
Glu	9.836 ± 1.372	10.552 ± 1.486	12.534 ± 1.815	11.966 ± 1.672
Gly	2.004 ± 0.283	2.320 ± 0.324	3.221 ± 0.454	3.177 ± 0.446
His	1.061 ± 0.015	1.158 ± 0.164	1.735 ± 0.238	1.609 ± 0.227
Ile *	1.754 ± 0.251	2.004 ± 0.282	2.983 ± 0.420	2.828 ± 0.398
Leu *	3.692 ± 0.512	4.038 ± 0.571	6.284 ± 0.885	5.797 ± 0.818
Lys *	1.743 ± 0.240	1.787 ± 0.251	2.121 ± 0.295	1.925 ± 0.271
Phe *	2.549 ± 0.359	2.808 ± 0.392	3959 ± 0.556	3.698 ± 0.517
Pro	2.320 ± 0.327	2.636 ± 0.370	3.063 ± 0.431	3.110 ± 0.435
Ser	2.287 ± 0.316	2.312 ± 0.324	3.913 ± 0.544	3.590 ± 0.502
Thr *	1.819 ± 0.251	1.737 ± 0.244	2.745 ± 0.386	2.412 ± 0.338
Tyr	1.514 ± 0.207	1.874 ± 0.263	3.698 ± 0.522	3.701 ± 0.517
Val *	2.571 ± 0.359	2.804 ± 0.392	4.378 ± 0.613	4.088 ± 0.557
Cis	1.015 ± 0.142	0.842 ± 0.118	1.146 ± 0.159	1.368 ± 0.193
Met *	0.797 ± 0.111	0.891 ± 0.126	1.554 ± 0.216	1.821 ± 0.256
Trp *	0.521 ± 0.052	0.556 ± 0.057	1.017 ± 0.102	0.996 ± 0.100

* Essential amino acids (EAAs).

## Data Availability

The data supporting the findings of this study are available within the article and in the Supplementary Materials (Figure S1). Raw reads were deposited in the Sequence Read Archive (SRA) database (BioProject accession number PRJNA1470175).

## Supplementary Materials

The following supporting information can be downloaded at https://www.mdpi.com/article/10.3390/foods15142442/s1, Figure S1: Principal Coordinate Analysis (PCoA) based on Bray–Curtis distances showing the β diversity of (A) bacterial and (B) yeast communities in oat and rice okara sourdoughs during fermentation. Samples are grouped according to fermentation time (T0, T1, T2) and matrix (oat in green, rice in yellow). Clear temporal shifts in community composition are observed in both matrices, leading to distinct clustering of samples at different fermentation stages. PERMANOVA analysis confirmed significant differences among groups (*p* = 1 × 10^−3^).
