# Supplementary material for: Unveiling Microbial Dynamics in the Spontaneous Fermentation of Oat and Rice Okara Sourdoughs
Source: Foods. 2026 Jul 9;15(14):2442. doi: 10.3390/foods15142442 (PMC13409664; doi:10.3390/foods15142442)
Supplement: Supplementary file 1 [file foods-15-02442-s001.zip › foods-4377343-supplementary.pdf]

Unveiling Microbial Dynamics in the Spontaneous Fermentation of Oat and Rice Okara Sourdoughs

Federica Meanti, Paolo Bellassi, Alessandra Fontana, Margherita Dall'Asta, Annalisa Rebecchi

Supplementary material

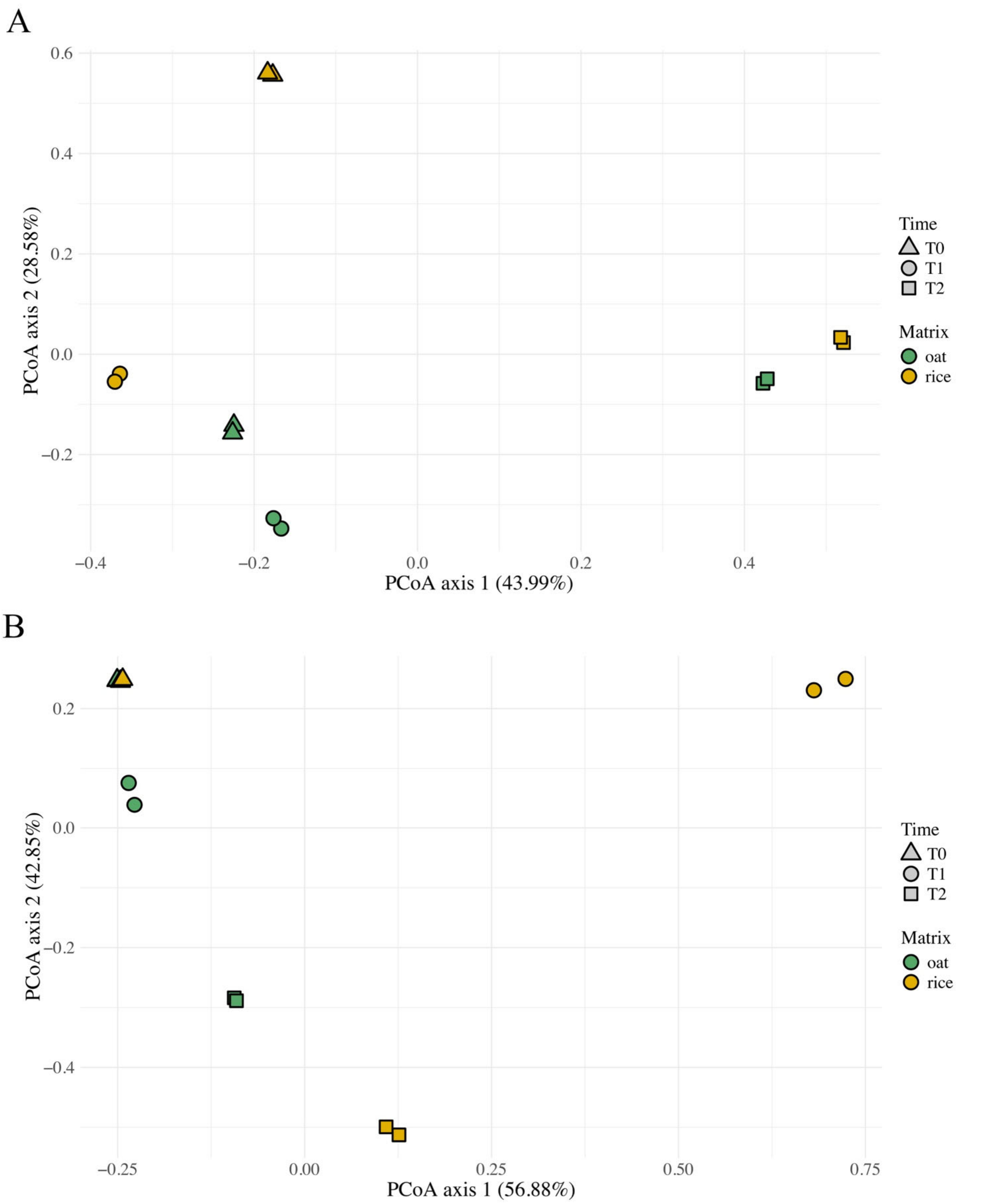

Figure S1. Principal Coordinate Analysis (PCoA) based on Bray–Curtis distances showing the β-diversity of (A) bacterial and (B) yeast communities in oat and rice okara sourdoughs during fermentation. Samples are grouped according to fermentation time (T0, T1, T2) and matrix (oat in green, rice in yellow). Clear temporal shifts in community composition are observed in both matrices, leading to distinct clustering of samples at different fermentation stages. PERMANOVA analysis confirmed significant differences among groups ($p = 1 \times 10^{-3}$).
